# Comprehensive microRNA expression analysis of pediatric gonadal germ cell tumors: unveiling novel biomarkers and signatures

**DOI:** 10.1002/1878-0261.13617

**Published:** 2024-05-09

**Authors:** Ana Glenda Santarosa Vieira, Luciane Sussuchi da Silva, Eduardo Caetano Albino da Silva, Ana Carolina Laus, Thaíssa Maria Veiga Faria, André van Helvoort Lengert, Gisele Eiras Martins, Marco Antônio de Oliveira, Rui Manuel Reis, Luiz Fernando Lopes, Mariana Tomazini Pinto

**Affiliations:** ^1^ Barretos Children's Cancer Hospital from Hospital de Amor Brazil; ^2^ Brazilian Childhood Germ Cell Tumor Study Group The Brazilian Pediatric Oncology Society (SOBOPE) São Paulo Brazil; ^3^ Pediatric Cancerology's Department of Santa Casa de Misericórdia de Santos Brazil; ^4^ Molecular Oncology Research Center Barretos Cancer Hospital Brazil; ^5^ Department of Pathology Barretos Cancer Hospital Brazil; ^6^ Life and Health Sciences Research Institute (ICVS), Medical School University of Minho Braga Portugal; ^7^ ICVS/3B's‐PT Government Associate Laboratory Braga Portugal; ^8^ Pediatric Oncology Research Group (GPOPed), Molecular Oncology Research Center Barretos Cancer Hospital Brazil

**Keywords:** biomarkers, germ cell tumor, microRNA, ovarian cancer, pediatric cancer, testicular cancer

## Abstract

microRNAs (miRNAs) are small endogenous noncoding RNAs, and alterations in their expression may contribute to oncogenesis. Discovering a unique miRNA pattern holds the potential for early detection and novel treatment possibilities in cancer. This study aimed to evaluate miRNA expression in pediatric patients with gonadal germ cell tumors (GCTs), focusing on characterizing the miRNA profiles of each histological subtype and identifying a distinct histological miRNA signature for a total of 42 samples of pediatric gonadal GCTs. The analysis revealed distinct miRNA expression profiles for all histological types, regardless of the primary site. We identified specific miRNA expression signatures for each histological type, including 34 miRNAs for dysgerminomas, 13 for embryonal carcinomas, 25 for yolk sac tumors, and one for immature teratoma, compared to healthy controls. Furthermore, we identified 26 miRNAs that were commonly expressed in malignant tumors, with six miRNAs (miR‐302a‐3p, miR‐302b‐3p, miR‐371a‐5p, miR‐372‐3p, miR‐373‐3p, and miR‐367‐3p) showing significant overexpression. Notably, miR‐302b‐3p exhibited a significant association with all the evaluated clinical features. Our findings suggest that miRNAs have the potential to aid in the diagnosis, prognosis, and management of patients with malignant GCTs.

## Graphical abstract

Germ cell tumors cover a spectrum of benign or malignant neoplasms in children that can present with different clinical presentations and in different locations. Discovering a unique miRNA pattern holds the potential for early detection and novel treatment possibilities. miRNA expression profiling reveals distinct signatures for each malignant histology of these tumors, offering applications in diagnosis, prognosis, and potential targeted therapy.

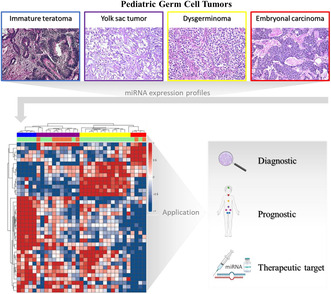

**Abbreviations**
AFP: alpha‐fetoprotein
AUC: area under the ROC curve
CC: choriocarcinoma
COG: Classification of The Children's Oncology Group
EAU: European Association of Urology
EC: embryonal carcinoma
FC: fold change
FFPE: formalin‐fixed paraffin‐embedded
FIGO: Classification of The International Federation of Gynecology and Obstetrics
GCTs: germ cell tumors
H&E: hematoxylin and eosin
HCG: human chorionic gonadotropin
IT: immature teratoma
LDH: lactic dehydrogenase
miRNA: microRNA
mRNAs: messenger RNAs
NSGCTs: nonseminomatous germ cell tumors
QC: quality controls
ROC: receiver operating characteristic curve
TGCT: testicular germ cell tumor
T‐SNE: t‐distributed stochastic neighborhood embedding
WHO: World Health Organization
YST: yolk sac tumor

## 1 Introduction

Germ cell tumors (GCTs) encompass a spectrum of benign or malignant neoplastic diseases that differ significantly in their clinical presentation, histology, and biological behavior [1, 2]. Among childhood tumors, GCTs are rare, accounting for approximately 3% of cases, and may arise either in gonadal or extragonadal sites [3].

Malignant GCTs are divided into seminomatous and nonseminomatous (NSGCTs). Seminomatous GCTs, resembling undifferentiated germ cells, are rare in young children and are more common in adolescents and young adults. Nonseminomatous GCTs can be further divided into different histologies, including embryonal carcinoma (EC), yolk sac tumor (YST), choriocarcinoma (CC), and immature teratoma (IT). Mature teratomas are generally benign and are characterized by well‐differentiated tissue. GCTs often exhibit mixed histology, encompassing EC, YST, CC, and teratoma components [4].

GCT diagnosis includes a physical examination, imaging modalities, and measurement of serum biomarkers [5]. A set of serum biomarkers are informative for diagnosis, risk assessment, and follow‐up, including alpha‐fetoprotein (AFP), human chorionic gonadotropin (HCG), and lactic dehydrogenase (LDH) [6]. AFP is primarily informative for YST with sporadic positivity in EC, while HCG is mainly informative for CC with sporadic positivity in EC [7]. LDH has been described as a serum marker of disease severity rather than a specific histological type [8]. Teratomas typically present absence in AFP and/or HCG serum levels [7]. However, none of these markers are specific for GCTs and may be expressed in other tumors such as hepatoblastoma, as well as non‐oncological conditions such as anatomical neural tube defect and diabetes, limiting their diagnostic and follow‐up utility. Therefore, more specific biomarkers are needed for accurate diagnosis, prognosis, and therapy monitoring in pediatric GCTs [6, 9]. Exploring miRNA signatures adapted to each GCT histological subtype is crucial.

microRNAs (miRNAs) are small endogenous noncoding RNAs consisting of 18–25 nucleotides [10]. MiRNAs play an important role in post‐transcriptional regulation by cleaving or repressing the translation of target messenger RNAs (mRNAs). Alterations in expression may contribute to oncogenesis by affecting cell proliferation, differentiation, and apoptosis, suggesting that miRNA alteration may lead to oncogenesis [11, 12].

Previous investigations have identified miRNAs associated with the tumorigenesis of GCTs. Palmer et al. [13] reported the overexpression of six miRNAs in malignant GCTs compared to normal tissues, demonstrating the association of these miRNAs with histological (YST/EC/seminoma), primary location (gonadal/extragonadal), and patient age (pediatric/adult). Syring et al. [14] found that serum levels of miRNAs were elevated in patients with testicular GCT (TGCT) and reported that the miR‐371a‐3p specifically outperformed HCG or AFP assays, suggesting the potential clinical utility of this miRNA in the management of TGCT patients. Furthermore, serum levels of miR‐371a‐3p presented superior performance compared to conventional markers (AFP, HCG, and LDH) [15].

Despite these studies, limited information is available regarding miRNA profiles that can distinguish between histological types of pediatric GCTs. Therefore, this study aimed to evaluate the miRNA expression in pediatric patients with gonadal GCTs, focusing on characterizing the miRNA profiles of each histological subtype and identifying a distinctive histological miRNA signature for pediatric gonadal GCTs.

## 2 Materials and methods

### 2.1 Study population and sample collection

This retrospective cohort study analyzed 42 pediatric gonadal GCT patients who were treated at Barretos Children's Cancer Hospital (Brazil), between 2000 and 2017. It included 31 GCT ovarian tumors and 11 testicular GCT (TGCT). Based on staining with hematoxylin and eosin (H&E), the slides from the paraffin block of each tumor resected at diagnosis had the tumor area delimited by the experienced pathologist to isolate the tumor material. Initially, a 5 micron cut was made in each paraffin block for H&E staining in order to delimit the tumor tissue. Next, five 10‐micron sections were made to be used in RNA extraction and, at the end, a new 5‐micron section was stained with H&E so that the pathologist could assess the permanence of the tumor in the previous sections. As a control group, we used non‐tumoral tissue adjacent to the tumors from 10 pediatric GCT patients. The classification of GCTs was determined according to the 2016 World Health Organization (WHO) guidelines. The risk of treatment was determined by risk stratification according to tumor stage. Stage I gonadal tumors were classified as low‐risk and were treated with surgery alone, without adjuvant chemotherapy. Stages II, III, and IV in patients younger than 11 years were classified as intermediate‐risk and received two chemotherapeutic agents (cisplatin and etoposide) for a total of four cycles. Stage IV gonadal tumors in patients aged 11 years or older were classified as high‐risk and received a total of five cycles of three chemotherapeutic agents (cisplatin, etoposide, and ifosfamide) and were followed up regularly with imaging and serologic markers [16].

Sociodemographic and clinicopathological data were retrospectively collected from medical records. This study was approved by the local ethics committee (Barretos Cancer Hospital IRB/Project No. 1692/2018). Due to the great difficulty in locating the participants to consent to them, even considering that a portion of this population has already died, the CEP‐HCB exempted the study from the application of the ICF based on if in your right by resolution 466/2012, believing that we could cause great emotional suffering for the family, in addition to being a retrospective study, which used only slides and paraffin blocks already stored in the pathology sector of the Barretos Cancer Hospital and consultation in medical records.

### 2.2 RNA isolation

RNA isolation was performed from formalin‐fixed paraffin‐embedded (FFPE) tumor samples, sectioned on slides with a thickness of 10 μm as previously reported [17]. Based on hematoxylin and eosin (H&E) staining, the area of tumor tissue was selected by an experienced pathologist for isolation of tumor material, containing at least 70% of the tumor area. RNA was isolated using the *RecoverAll Total Nucleic Acid Isolation Kit* (Thermo Fisher Scientific, Waltham, MA, USA), according to the manufacturer's instructions. The purity and quantification of total RNA were assessed using a ND‐1000 NanoDrop spectrophotometer (Thermo Fisher Scientific) and a Qubit 2.0 instrument (Thermo Fisher Scientific).

### 2.3 NanoString nCounter system for miRNA expression assessment

The expression of approximately 800 human miRNAs was measured using the Human v3 miRNA Assay CSO Panel (CSO‐MIR3‐12, NanoString Technologies, Seattle, WA, USA) with the nCounter Analysis System technology (NanoString Technologies) as previously reported [18]. Sample preparation, hybridization, detection, and scanning procedures were performed according to the instructions of NanoString Technologies (NanoString). Briefly, total RNA (100 ng), quantified by Qubit Fluorometer System (Thermo Fisher Scientific), was initially submitted to the miRNA tag ligation reaction, followed by hybridization with Capture and Reporter Probes for 21 h at 65 °C. The purification and immobilization of probe–target complexes were performed in the nCounter PrepStation System and the cartridges were analyzed in nCounter Digital Analyzer (NanoString), which captures up to 555 fields of view per sample. Standardized quality controls (QC) were performed using nsolver software (NanoString) for all samples, including imaging QC, binding density, limit of detection, positive controls linearity, and negative control levels. All samples that met quality control criteria were eligible for data analysis. Data analysis was performed using R v4.1.2 (The R Foundation, Viena, Austria) with the nanostringnorm package v 1.2.1.1 [19, 20]. Evaluated miRNAs were considered not expressed if their raw expression level was below the mean plus five times the standard deviation of the negative controls in all samples. Normalization of samples was performed using the quantile method and log_2_ transformation, followed by differential expression and statistical analysis. For differential expression, a two‐fold change (FC) difference in miRNA expression levels and a significance level of Bonferroni‐corrected *P*‐value ≤ 0.05 were adopted.

### 2.4 Statistical analysis

Heatmaps and clustering of differentially expressed genes were generated with the complex heatmap r package v2.12.1 [18] using Pearson's correlation distance. The R implementation of t‐distributed stochastic neighbor embedding [21, 22] in the rtsne package v0.15 was applied to visualize the histological subgroups of GCTs. Boxplots of gene expression levels and Kruskal–Wallis tests were created using the ggpubr package v0.4.0.

Statistical analyses associated with clinical factors were performed using the ibm‐spss software program for Windows/Mac, version 23.0 (SPSS Inc, Chicago, IL, USA). A *P*‐value ≤ 0.05 was considered statistically significant.

Samples were characterized using frequency tables for qualitative variables, and central tendency (mean, median) and dispersion (standard deviation, minimum, and maximum) were measured for quantitative variables.

The Student's *t*‐test or Mann–Whitney test was performed after verification of normality, which was tested by the Kolmogorov–Smirnov test. The ROC curve analysis was performed using the rocr package v1.0‐11 [21]. MicroRNAs presenting an area under the ROC curve (AUC) ≥ 0.75 were considered to have a good performance.

## 3 Results

### 3.1 Clinicopathologic features of pediatric GCT patients

The clinicopathologic features of the 42 patients are summarized in Table 1. Most patients had ovarian tumors (*n* = 31, 73.8%), and 11 patients (26.2%) had testicular tumors. The average age at diagnosis was 11.8 years (range, 0–19 years), with females 14.1 years and males 5.4 years old. Clinical findings showed that 26.2% of patients (11/42) had yolk sac tumors, followed by 16.7% mature teratomas and 9.5% embryonal carcinomas. Among female patients, 16% (5/31) had immature teratomas and 42% had dysgerminomas (13/31), while among male patients, 18.2% (2/11) had prepubertal teratomas.

**Table 1:** Clinicopathologic features of GCTs in pediatric patients. COG, classification of The Children's Oncology Group; DP, standard deviation; FIGO, classification of The International Federation of Gynecology and Obstetrics.

Features	Total, *n* (%)	Ovary, *n* (%)	Testis, *n* (%)
*N* (%)	42 (100)	31 (73.8)	11 (26.2)
Average age (years)	11.8 (5.4)	14.1 (2.9)	5.4 (6.0)
Histology			
Yolk sac tumor	11 (26.2%)	4 (13%)	7 (63.6%)
Embryonal carcinoma	4 (9.5%)	2 (6.5%)	2 (18.2%)
Mature teratoma	7 (16.7%)	7 (22.5%)	–
Immature teratoma	5 (11.9%)	5 (16%)	–
Morris grade I	1	1	–
Morris grade II	2	2	–
Morris grade III	2	2	–
Prepubertal testicular teratoma	2 (4.7%)	–	2 (18.2%)
Postpubertal testicular teratoma	0	–	0
Dysgerminoma/Seminoma	13 (31%)	13 (42%)	0
Staging			
FIGO classification			
I	15	15 (48.3%)	–
II	4	4 (13%)	–
III	10	10 (32.2%)	–
IV	2	2 (6.5%)	–
COG classification			
I	13	5 (16.1%)	8 (72.8%)
II	12	11 (35.5%)	1 (9%)
III	13	13 (41.9%)	0 (0)
IV	4	2 (6.5%)	2 (18.2%)
Risk			
Low	21 (50%)	13 (41.9%)	8 (72.8%)
Intermediate	8 (19%)	7 (22.6%)	1 (9%)
High	13 (31%)	11 (35.5%)	2 (18.2%)
Metastasis			
Yes	10 (23.8%)	7 (22.5%)	3 (27.3%)
No	32 (76.2%)	24 (77.5%)	8 (72.7%)
Chemotherapy			
Yes	21 (50%)	18 (58%)	3 (27.3%)
No	21 (50%)	13 (42%)	8 (72.7%)
Relapse			
Yes	5 (12%)	2 (6.5%)	3 (27.3%)
No	37 (88%)	29 (93.5%)	8 (72.7%)
Status			
Alive	39 (92.8%)	29 (93.5%)	10 (91%)
Dead	3 (7.2%)	2 (6.5%)	1 (9%)

Around half of the patients were classified as low‐risk (50%) and had early stages (FIGO I/COG I) (48.3%), and 23.8% (10/42) had metastases at diagnosis. Half of the patients were treated with chemotherapy, a minority had tumor recurrence (12%), and 92.8% were still alive.

### 3.2 microRNA expression signature of the individual histological types of GCTs

**Fig. 1:**
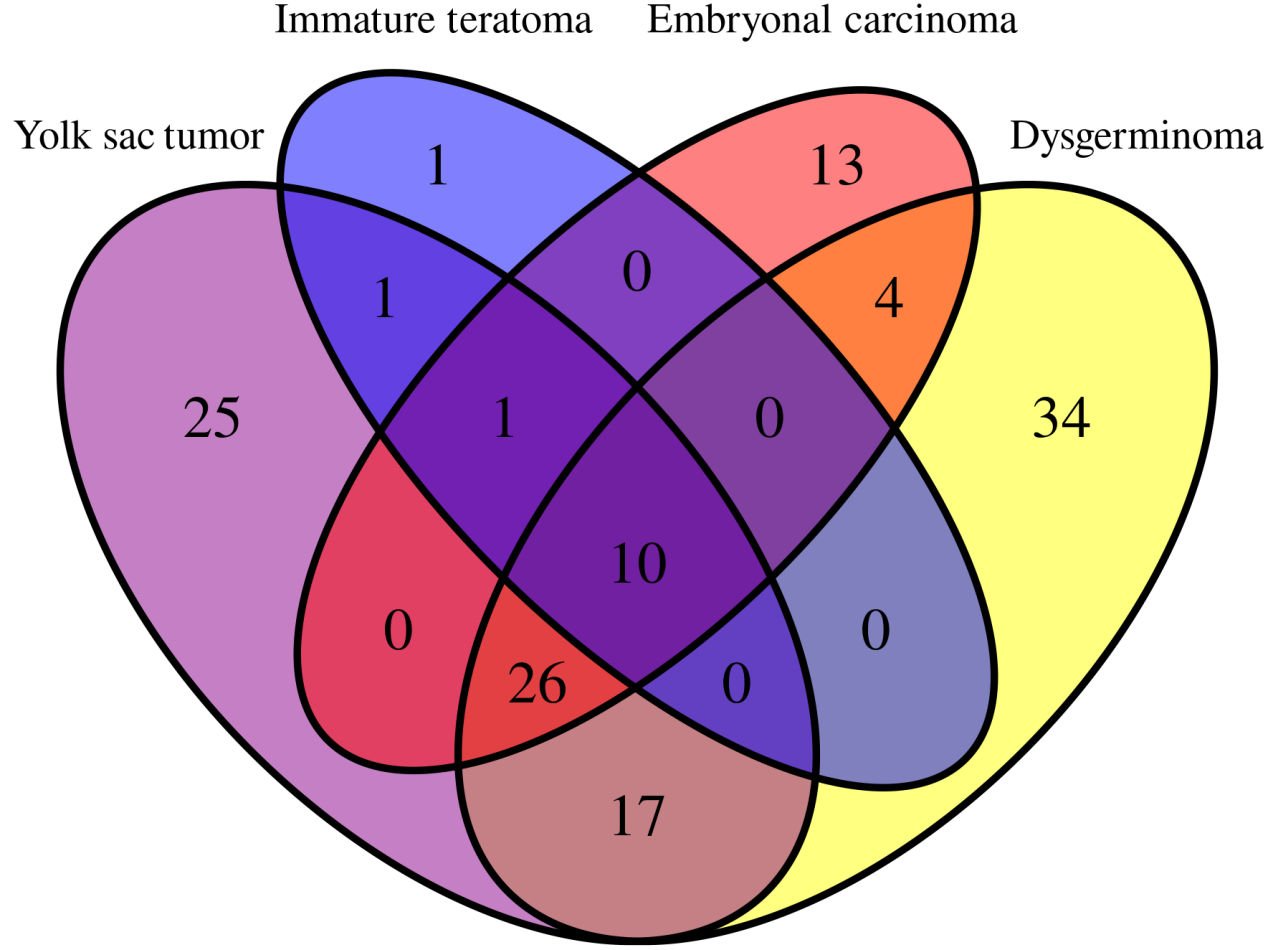
Venn diagram shows miRNA distribution among the different GCT histologies. Venn diagrams indicating the number of significant differentially expressed miRNAs in embryonal carcinomas (13 miRNAs), dysgerminomas (34 miRNAs), yolk sac tumors (25 miRNAs), and immature teratomas (1 miRNA), and the overlap regions showing the number of miRNAs that are expressed in two or more histologies.

All 42 cases showed reliable miRNA analysis. Initially, we examined the miRNA profiles of both ovarian and testicular tumors. As expected, we found that mature teratomas clustered with healthy controls (Fig. S1). Subsequently, we examined the miRNA profiles of malignant tumors of both sites individually. The miRNA expression profile of each histology was compared with healthy controls to identify differentially expressed miRNAs for each histological type (Fig. 1 and Fig. S2).

We identified 91 differentially expressed miRNAs in dysgerminomas compared to healthy controls, with 34 specifics to this histological subtype, of which 30 miRNAs were upregulated and four downregulated (Table S1). Fifty‐four miRNAs were differentially expressed between the embryonal carcinoma and controls, with 13 being specific to EC. Among these 13 miRNAs, one was upregulated and 12 were downregulated (Table S2). Yolk sac tumors showed 80 differentially expressed miRNAs compared to healthy controls. Among these, 25 miRNAs were specific to YST, with 20 upregulated and five downregulated (Table S3). When immature teratomas were compared with healthy controls, we observed 13 differentially expressed miRNAs, of which only one (miR‐199b‐5p) (*P*_adj = 0.0013 and log_2_ fold change of 3.1) was upregulated and specific for immature teratomas.

**Fig. 2:**
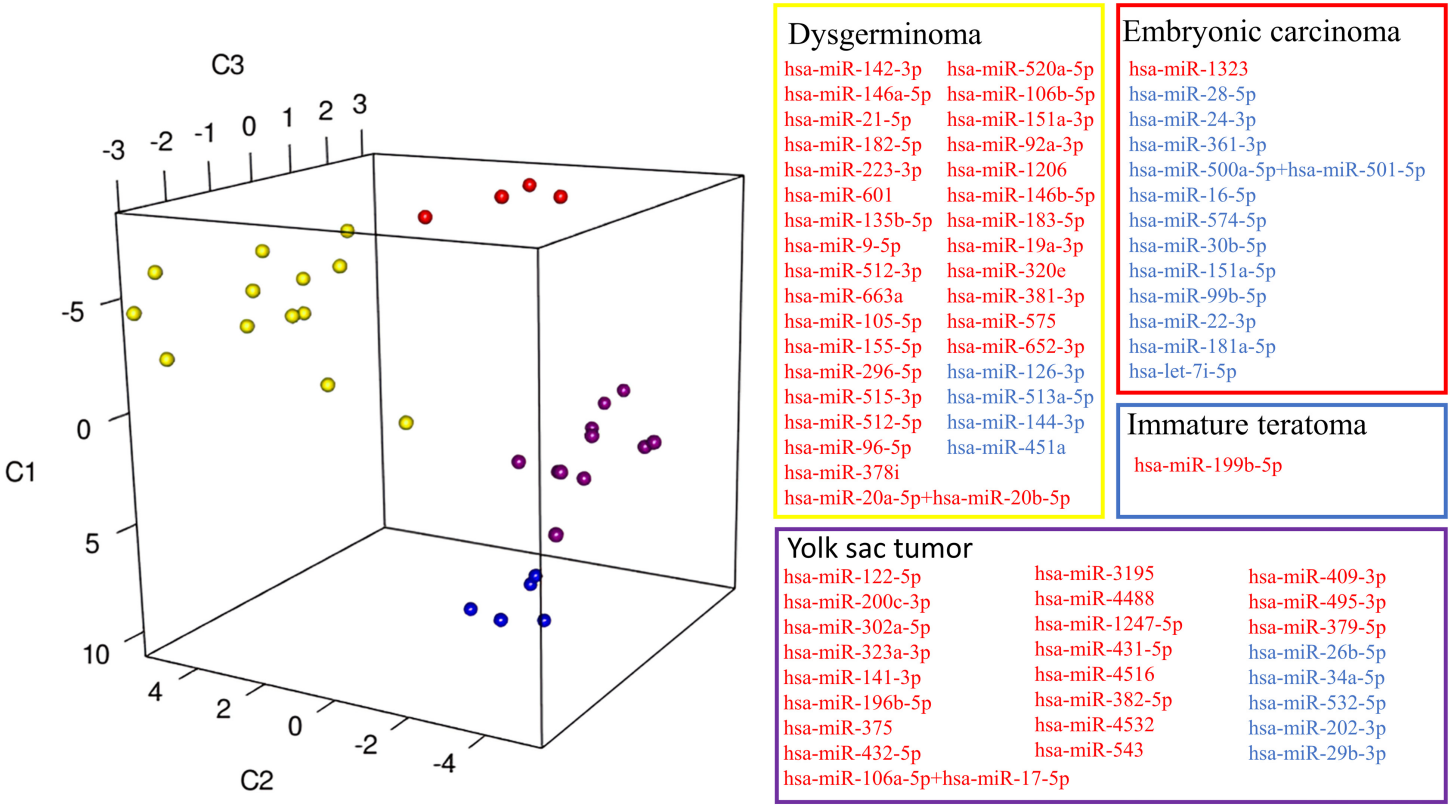
T‐SNE visualization of miRNA expression according to GCT histological types. Embryonal carcinoma, yolk sac tumor, dysgerminoma, and immature teratoma express specific signatures of miRNAs. Data points (representing patients) have been color‐coded based on their histologies, and miRNAs have been grouped together based on the same histological patterns. Yellow dots indicate dysgerminoma samples, red dots the embryonal carcinoma, purple dots the yolk sac tumors, and blue dots immature teratoma. Blue and red colors indicate the miRNAs with lower and higher expression levels, respectively.

The t‐distributed stochastic neighborhood embedding (T‐SNE) algorithm was further used to visualize the classification of different histological types of GCTs based on the normalized expression of the histology‐specific miRNA (Fig. 2). Our results show each histological type expressing a specific miRNA signature.

### 3.3 Overlap of differentially expressed miRNAs in pediatric malignant tumor samples

To analyze the similarity of miRNA expression among the different malignant histological types, we evaluated their overlap. The analysis revealed a total of 132 unique differentially expressed miRNAs across the four malignant histological types (Fig. 1). Only hsa‐miR‐205‐5p showed an overlap between YST and IT. The hsa‐miR‐200b‐3p was common to YST, IT, and EC, while four miRNAs showed an overlap between EC and dysgerminoma (miR‐1246, miR‐135a‐5p, miR‐140‐5p, and miR‐342‐3p). In addition, 17 miRNAs were common to dysgerminomas and YST (let‐7b‐5p, miR‐10b‐5p, miR‐130a‐3p, miR‐150‐5p, miR‐1915‐3p, miR‐194‐5p, miR‐199a‐3p + hsa‐miR‐199b‐3p, miR‐199a‐5p, miR‐23b‐3p, miR‐25‐3p, miR‐302c‐3p, miR‐302d‐3p, miR‐509‐5p, miR‐514b‐5p, miR‐596, miR‐7‐5p, and miR‐93‐5p).

Among all malignancies, 10 miRNAs in common were downregulated when compared to normal tissues, including miR‐195‐5p, miR‐29a‐3p, miR‐497‐5p, miR‐503‐5p, miR‐506‐3p, miR‐507, miR‐509‐3p, miR‐513b‐5p, miR‐513c‐5p, and miR‐514a‐3p. The analysis also revealed an overlap of 26 miRNAs between YST, EC, and dysgerminomas, with 20 miRNAs downregulated and six upregulated in tumor tissues (Table 2).

**Table 2:** Overlap of 26 differentially expressed miRNAs in yolk sac tumor, dysgerminoma, and embryonal carcinoma.

miRNAs	Yolk sac tumor		Dysgerminoma		Embryonal carcinoma	
	*P*_adj	Log_2_ fold change	*P*_adj	Log_2_ fold change	*P*_adj	Log_2_ fold change
miR‐302b‐3p	5.2E‐11	9.3	9,9E‐09	6.4	0.003	7.9
miR‐302a‐3p	1.6E‐10	7.5	2.5E‐08	3.8	0.006	6.1
miR‐372‐3p	2.0E‐08	6.5	4.8E‐15	7.5	0.00027	6.6
miR‐373‐3p	2.6E‐07	6.0	4.8E‐15	7.5	1.0E‐07	6.4
miR‐367‐3p	1.6E‐10	5.8	1.3E‐05	2.6	0.049	4.9
miR‐371a‐5p	0.0014	1.8	1.5E‐10	3.5	0.0012	3.3
let‐7e‐5p	0.0046	−1.0	1.1E‐05	−1.9	0.003	−3.5
miR‐27b‐3p	0.00034	−1.0	1.1E‐05	−1.3	0.0031	−2.0
miR‐214‐3p	0.011	−1.1	0.0015	−1.7	0.006	−2.6
let‐7d‐5p	0.00059	−1.3	6.3E‐08	−1.7	0.025	−3.8
let‐7f‐5p	0.0021	−1.3	0.00086	−1.4	1.1E‐06	−3.1
miR‐98‐5p	3.3E‐06	−1.4	1.4E‐05	−1.6	3.8E‐06	−2.6
miR‐26a‐5p	0.0023	−1.5	0.025	−1.0	0.0028	−3.0
miR‐29c‐3p	0.0013	−1.7	0.041	−1.0	0.00052	−3.9
miR‐145‐5p	0.0021	−1.9	0.00048	−2.2	0.0014	−4.8
let‐7a‐5p	0.00048	−2.0	0.0041	−1.8	0.035	−10
miR‐450a‐5p	8.9E‐05	−2.0	4.5E‐08	−3.1	0.018	−3.3
miR‐204‐5p	0.013	−2.1	0.00075	−2.9	0.047	−2.3
let‐7 g‐5p	6.1E‐06	−2.2	0.0037	−1.1	0.002	−3.7
miR‐509‐3‐5p	0.0065	−2.5	0.006	−2.6	0.011	−2.9
miR‐100‐5p	5.6E‐07	−2.7	1.5E‐05	−1.9	0.0037	−3.8
miR‐424‐5p	6E‐07	−2.8	1.5E‐11	−4.4	0.048	−4.8
let‐7c‐5p	1.5E‐05	−3.4	4.4E‐09	−3.6	0.000039	−5.3
miR‐125b‐5p	2.8E‐06	−3.5	2.3E‐06	−2.7	0.0051	−5.9
miR‐99a‐5p	6.7E‐08	−3.7	9.9E‐09	−3.9	0.000039	−5.3
miR‐508‐3p	0.00051	−4.8	0.00038	−5.1	0.024	−4.4

**Fig. 3:**
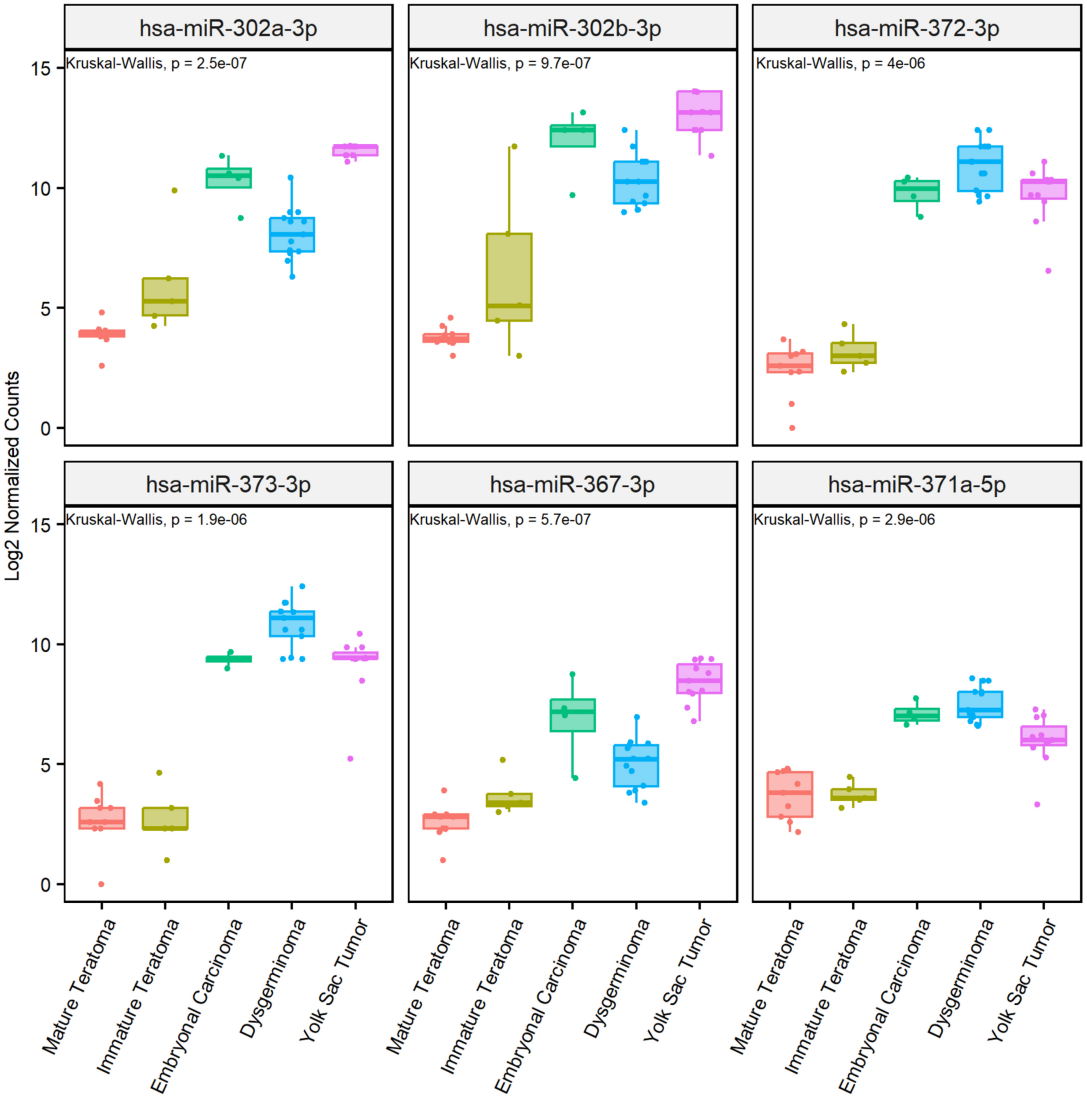
MiRNA expression levels in different histological types of pediatric GCT patients. Box‐plot expression of miR‐302a‐3p, miR‐302b‐3p, miR‐372‐3p, miR‐373‐3p, miR‐367‐3p, and miR‐371a‐5p in dysgerminoma (*n* = 13), embryonal carcinoma (*n* = 4), mature (*n* = 7) and immature teratomas (*n* = 5), and yolk sac tumors (*n* = 11).

We further compared the six overexpressed miRNAs in malignant GCT (Table 2) across all histologies (Fig. 3). Different histologies showed a distinct profile of miRNA expression. Notably, yolk sac tumors, which are associated with greater tumor aggressiveness, exhibited overexpression of miR‐302a‐3p and miR302b‐3p (*P* ≤ 0.001) compared to the other histologies, suggesting that these two miRNAs could be used to evaluate the prognosis of GCT patients.

**Fig. 4:**
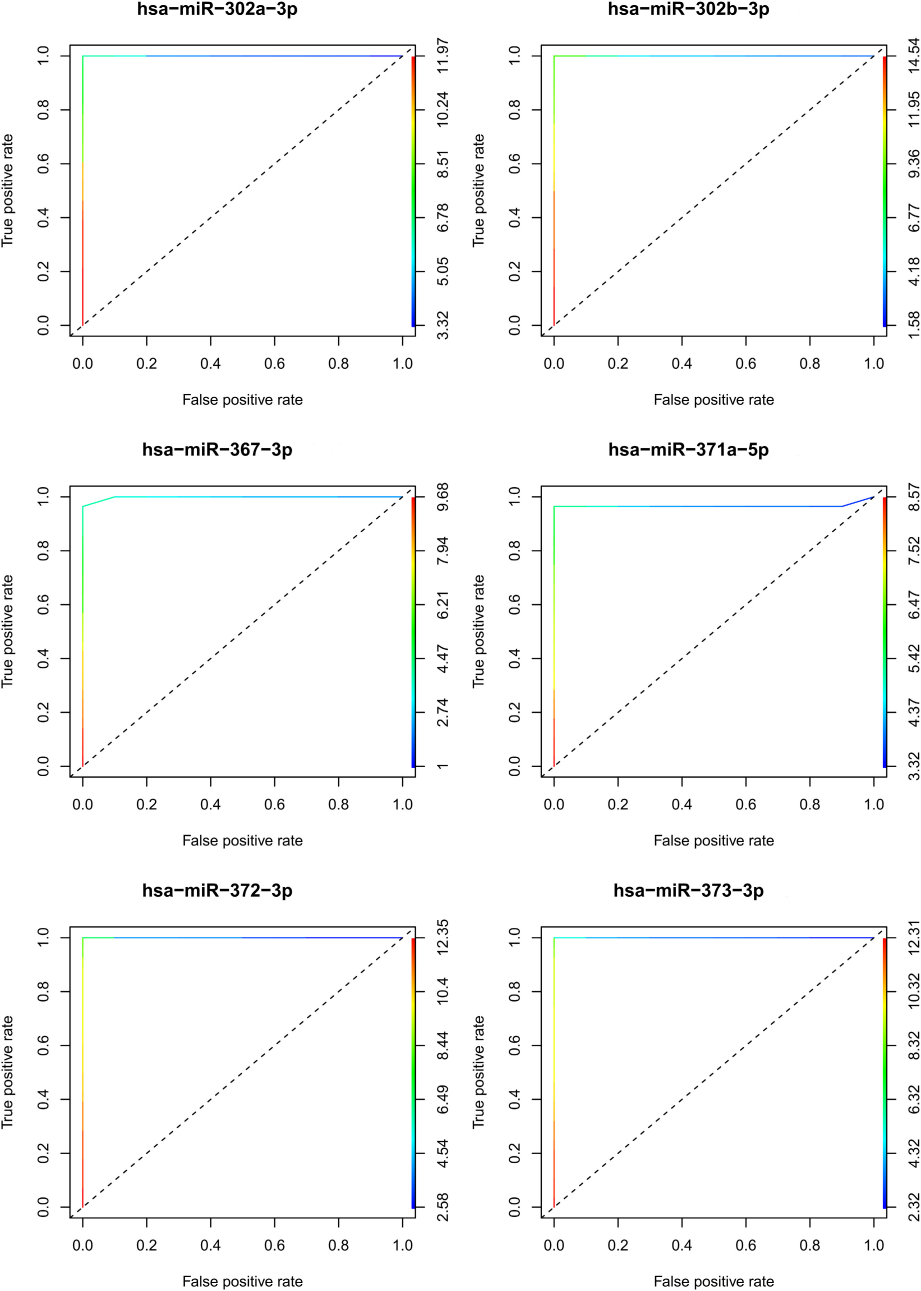
Individual ROC curves for differentially expressed miRNAs in pediatric GCT patients. ROC curves show the performance of the six miRNAs in malignant GCTs (embryonal carcinoma, yolk sac tumor, dysgerminoma, and immature teratoma), including miR‐302a‐3p, miR‐302b‐3p, miR‐367‐3p, miR‐371a‐5p, miR‐372‐3p, and miR‐373‐3p. AUC, area under the curve and ROC, receiver operating characteristic.

To further assess the relevance of these six miRNAs, a ROC curve was established, and the AUC was determined (Fig. 4). All six miRNAs exhibited a significant AUC value (1.0), suggesting their significant diagnostic value for discrimination between control and GCT pediatric patients. Further studies with larger sample sizes are necessary to confirm their predictive value.

Next, we analyzed the association of these miRNAs with clinical characteristics, including histology, risk, metastasis, AFP marker before surgery, relapse, and status (Table 3). We observed that miR‐302b‐3p was the only miRNA significantly associated with all the evaluated parameters, followed by miR‐302a‐3p, which showed significant association with all parameters except for metastasis. Due to the small number of cases, it was impossible to perform a survival analysis; however, patients who died had a significantly higher expression of miR‐302a‐3p (*P* = 0.009) and miR‐302b‐3p (*P* = 0.036). MiR‐367‐3p was significantly associated with AFP marker before surgery (*P* = 0.002), and miR‐371a‐5p was significantly associated with risk (*P* ≤ 0.001). No significant associations were observed for miR‐372‐3p and miR‐373‐3p.

**Table 3:** Correlation between differentially expressed miRNAs and clinical features of pediatric GCT patients with yolk sac tumor, dysgerminoma, and embryonal carcinoma. AFP, alpha‐fetoprotein; GCTs, germ cell tumors; Max, maximum; Min, minimum.

Feature	hsa‐miR‐302a‐3p		hsa‐miR‐302b‐3p		hsa‐miR‐367‐3p		hsa‐miR‐371a‐5p		hsa‐miR‐372‐3p		hsa‐miR‐373‐3p	
	Median (min; max)	*P* value	Median (min; max)	*P* value	Median (min; max)	*P* value	Median (min; max)	*P* value	Median (min; max)	*P* value	Median (min; max)	*P* value
Histology												
Yolk sac tumor	11.8 (11.1; 11.8)	**< 0.001** ^**a**^	13.3 (11.5; 13.8)	**< 0.001** ^**a**^	8.5 (6.7; 9.5)	**< 0.001** ^**a**^	5.8 (3.3; 7.3)	**< 0.001** ^**a**^	10.1 (6.5; 11.1)	0.061^a^	9.5 (5.1; 10.5)	**< 0.001** ^**a**^
Embryonal carcinoma	10.3 (2.3; 4.6)		11.6 (9.6; 13.1)		7.3 (4.2; 8.5)		7.1 (6.6; 7.8)		9.6 (8.7; 10.3)		9.3 (8.8; 9.6)	
Mature teratoma	3.7 (2.3; 4.6)		3.6 (3.0; 4.5)		–		3.5 (2.0; 4.7)		–		–	
Immature teratoma	5.1 (4.3; 9.9)		5.0 (3.0; 11.6)		3.4 (2.5; 5.0)		–		–		2.5 (0; 4.6)	
Dysgerminoma	7.9 (6.2; 10.3)		9.9 (8.7; 11.7)		5.1 (3.4; 6.9)		7.2 (6.5; 8.3)		10.9 (9.5; 11.6)		10.9 (9.4; 11.6)	
Risk												
Low	4.6 (2.3; 11.8)	**0.050** ^**a**^	5.0 (3.0; 13.7)	**0.041** ^**a**^	6.1 (2.5; 8.9)	0.854^a^	5.1 (2.0; 7.8)	**< 0.001** ^**a**^	10.1 (8.5; 11.6)	0.642^a^	9.5 (0; 11.4)	0.732^a^
Intermediate	9.3 (6.2; 11.5)		11.5 (8.2; 13.3)		5.4 (3.8; 9.3)		7.6 (5.9; 7.9)		10.1 (9.7; 11.4)		9.5 (3.1; 11.2)	
High	8.6 (5.1; 11.8)		10.9 (4.3; 13.7)		5.8 (3.4; 9.5)		7.0 (3.3; 8.3)		10.5 (6.5; 11.6)		9.9 (2.5; 11.6)	
Metastasis												
Yes	10.1 (5.1; 11.8)	0.128^b^	11.2 (4.4; 13.3)	**< 0.001** ^**b**^	6.3 (3.4; 9.5)	0.603^b^	7.1 (3.3; 8.3)	0.073^b^	10.3 (6.5; 11.6)	0.784^b^	9.5 (2.5; 11.6)	0.802^b^
No	7.8 (2.3; 11.8)		9.5 (3.0; 13.8)		5.7 (2.5; 9.4)		5.9 (2.0; 8.2)		10.2 (8.5; 11.6)		9.5 (0; 11.4)	
AFP pre surgical												
Normal	6.8 (2.3; 11.8)	**0.001** ^**b**^	8.8 (3.0; 12.6)	**< 0.001** ^**b**^	5.1 (3.1; 7.9)	**0.002** ^**b**^	6.5 (2.0; 8.3)	0.711^b^	10.8 (6.5; 11.6)	0.573^b^	10.9 (2.5; 11.6)	0.351^b^
Increased	11.6 (6.9; 11.8)		13.3 (9.6; 13.7)		8.2 (5.6; 9.5)		5.9 (5.5; 7.3)		10.2 (9.5; 11.1)		9.5 (9.4; 10.5)	
Relapse												
No	7.7 (2.3; 11.8)	**0.016** ^**b**^	9.6 (3.0; 13.7)	**0.029** ^**b**^	5.6 (2.5; 9.5)	0.116^b^	6.6 (2.2; 8.3)	> 0.999^b^	10.3 (6.5; 11.6)	0.413^b^	9.5 (0; 11.6)	0.942^b^
Yes	11.5 (8.5; 11.8)		12.6 (9.6; 13.7)		7.9 (4.2; 9.4)		5.9 (5.1; 7.8)		10.1 (8.5; 11.1)		9.5 (8.5; 10.5)	
Status												
Alive	7.9 (2.3; 11.8)	**0.009** ^**b**^	9.6 (3.0; 13.7)	**0.036** ^**b**^	5.6 (2.5; 9.5)	0.115^b^	6.7 (2.0; 8.3)	0.202^b^	10.3 (8.7; 11.6)	0.219^b^	9.5 (0; 11.6)	0.491^b^
Dead	11.8 (11.5; 11.8)		12.6 (12.6; 13.7)		7.9 (6.7; 9.4)		5.1 (3.3; 5.9)		8.5 (6.5; 11.1)		8.5 (5.1; 10.5)	

^a^ *Kruskal–Wallis* test.

^b^ *Mann–Whitney* test.

All numbers in bold are events that had significance with a *P* value < 0.05, according to the Materials and Methods section, item 2.4 statistical analysis.

### 3.4 Identification of differentially expressed miRNAs among pediatric malignant tumors

**Fig. 5:**
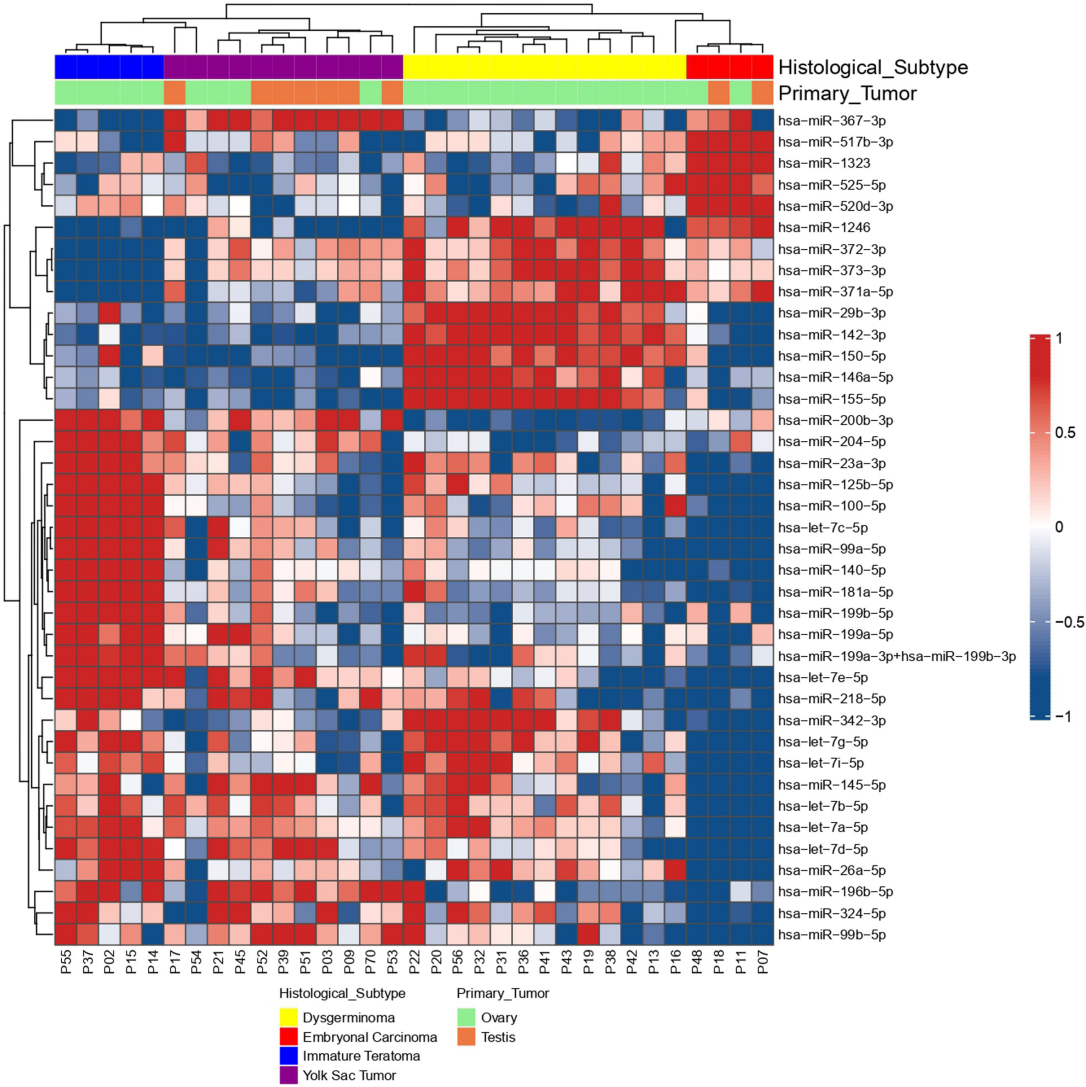
Heatmap and dendrogram of differentially expressed miRNAs in different histological types. Expression levels were used to group miRNA profiles according to their similarities between histological types, including embryonal carcinoma (red), dysgerminoma (yellow), yolk sac tumor (purple), and immature teratoma (blue). The heatmap shows a distinct miRNA expression profile in the four groups studied. Rows indicate the relative expression levels for a single miRNA, and columns indicate the expression level for a single sample. Blue and red colors indicate the miRNAs with lower and higher expression levels, respectively.

We found significant clustering of miRNA expression profiles according to histological type (Fig. 5). Two main clusters were observed: (a) YST and immature teratomas and (b) dysgerminomas and EC. The analysis also revealed a clear separation between the different histological subtypes (Fig. 5). In total, we identified 39 miRNAs with differential expression in the malignant tumor samples, namely miR‐367‐3p and miR‐196b‐5p were associated with YST; the miR‐371~373 cluster, miR142‐3p, and miR‐146a‐5p were associated with dysgerminomas; miR‐517b‐3p, miR‐1323, miR‐525‐5p, and miR‐99b‐5p were associated with EC; and miR‐199a‐5p, miR‐199b‐5p, miR‐125b‐5p, and let‐7c/e were associated with immature teratomas.

**Fig. 6:**
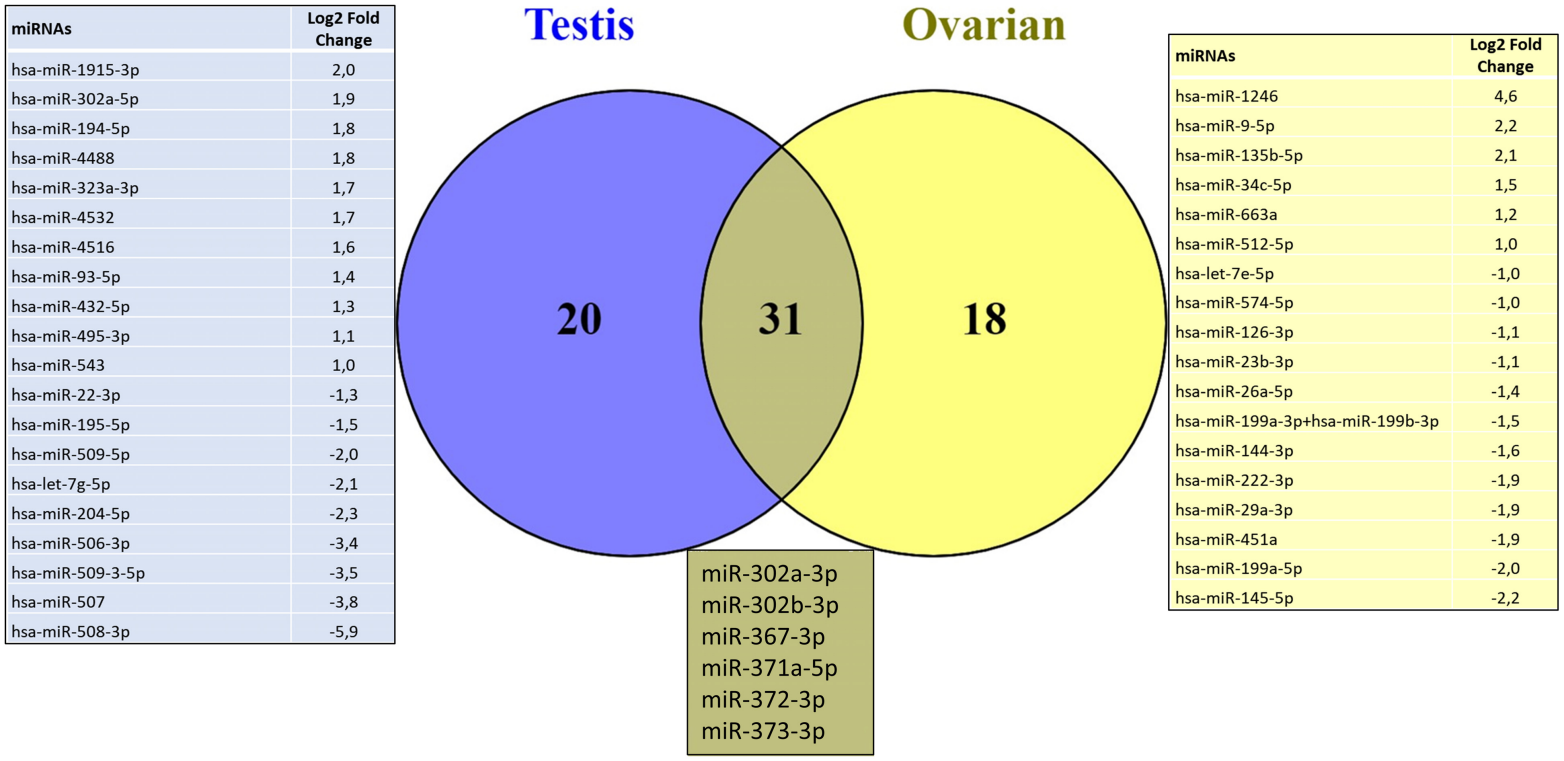
Venn diagram shows miRNA distribution among the testis and ovarian tumors. Venn diagrams indicate the number of significant differentially expressed miRNAs in testis (20 miRNAs) and ovarian (18 miRNAs) tumors, and the overlap regions show the number of miRNAs expressed in two tumors.

Since each histology presented a signature of miRNAs, we investigated whether this signature could change according to the primary location of the tumor. Therefore, we evaluated the common and specific miRNAs between the ovary and testis (Fig. 6, Table S4). We identified 20 miRNAs specific to testicular tumors (11 miRNAs upregulated and nine downregulated) and 18 to ovarian tumors (six miRNAs upregulated and 12 downregulated). In addition, 31 differentially expressed miRNAs were in common between the ovary and testis, in which the six miRNAs (miR‐302a‐3p, miR‐302b‐3p, miR‐367‐3p, miR‐371a‐5p, miR‐372‐3p, and miR‐373‐3p) were the most upregulated.

## 4 Discussion

In the present study, miRNA expression was analyzed in a series of pediatric patients with gonadal GCTs. Initially, we identified differentially expressed miRNAs that could determine a specific signature of different histological types of GCTs, irrespective of the primary site (ovary or testicle). Furthermore, we identified miRNAs that were commonly expressed in malignant tumors and exhibited significant associations with the clinical features evaluated.

The age distribution of the patients in our study aligns with previous studies on pediatric GCTs, which exhibit a bimodal distribution with peaks in children under 5 years of age and in adolescence [23]. Yolk sac tumors and teratomas, or a combination of these two tumors (mixed tumors), are more frequent in younger children, while seminomas/dysgerminomas are predominant in pubertal and young adult patients [4]. Our results also showed patient relapse percentages consistent with the European Association of Urology (EAU) guidelines for testicular cancer, in which 15–30% of patients with TCGTs will relapse after first‐line chemotherapy and require additional therapies. The low number of cancer‐related deaths in our series is consistent with the high overall survival rate of GCTs, which is above 90% [24].

Evaluating the miRNA expression profiles in cancer may allow insights into tumorigenesis pathways since tumors present specific miRNA signatures [25]. The potential of miRNA expression as a tool to assist the diagnosis, prognosis, and management of patients with GCTs has been reported [13, 26, 27]. However, most studies are not specific to pediatric patients. Here, we analyzed 42 pediatric patients with GCTs, including primary tumors from ovaries and testicles, and different histological types. In this study, we excluded cases of mixed tumors to focus on identifying specific miRNA signatures for each histological type. In our preliminary analysis, as expected, the mature teratoma clustered with healthy control samples, indicating that patients with mature teratomas have similar molecular profiles of controls and may explain the fact that they are usually asymptomatic and have an overall good prognosis [28, 29]. All mature teratoma samples in our study were ovarian tumors, and previous studies have described that teratoma outside of the ovary is rare, and mature cystic teratoma is the most common GCT occurring in the ovary (90% in premenarchal girls and 60% in individuals younger than 20 years old) [28, 29]. Furthermore, we showed that malignant histological types were grouped according to their miRNA expression profile, regardless of the primary site, suggesting that miRNA expression may be associated with the aggressive behavior of each GCT histological type.

Although we found an overlap of 26 miRNAs in the malignant tumors (yolk sac tumor, dysgerminoma, embryonal carcinoma, and immature teratoma), only six miRNAs were overexpressed (miR‐302a‐3p, miR‐302b‐3p, miR‐371a‐5p, miR‐372‐3p, miR‐373‐3p, and miR‐367‐3p). Notably, miR‐302b‐3p showed a significant correlation with all the evaluated clinical features, followed by miR‐302a‐3p, except for metastasis. Similar results were reported by Palmer et al., which demonstrated that the miR‐371~373 and miR‐302 clusters were differentially expressed regardless of histological subtype, site, or patient age [13]. Another study identified the miR‐371~373 and miR‐302 clusters as potential serum markers for malignant GCTs [30]. Special emphasis has been placed on the miRNA‐371a‐3p, which in a multicentric study has shown high sensitivity and specificity in the serum of patients with TGCTs, with an area under the curve (AUC) of 0.96 [31, 32]. In addition, circulating miR‐371a‐3p was suggested as a biomarker for adult patients with TGCT prior to orchiectomy [15]. All these data support the use of miR‐371a‐3p in GCT patients, especially in adults with TGCTs. Interestingly, our results suggest that for pediatric patients with GCT, regardless of the primary tumor location, the miR‐302a‐3p and miR‐302b‐3p present greater importance and potential as biomarkers. The evaluation of the standard tumor markers AFP and HCG in malignant GCTs is limited due to their restricted sensitivity and specificity, and a significant number of patients will be negative for these markers. Thus, it is important to identify specific differentially expressed miRNAs for each histological type. In our study, we found 34 miRNAs specific for dysgerminomas when compared with healthy control samples, 13 miRNAs specific to EC, 25 miRNAs specific to YST, and one miRNA specific to immature teratoma. This is the first time that a comprehensive list of differentially expressed miRNAs has been described for each histological type of GCTs. In the immature teratoma analysis, miR‐199b‐5p was overexpressed, and similar results were reported in pediatric intracranial GCTs [33]. In medulloblastoma, miR‐199b‐5p was associated with metastasis [34]. Most of the miRNAs identified in our study were overexpressed in tumor tissues, except for EC, which presented only one miRNA upregulated (miR‐1323) and 12 were downregulated. To the best of our knowledge, miR‐1323 has not been previously reported in GCTs, although it has been associated with tumor progression in hepatocellular carcinoma [35], migration in lung adenocarcinoma [36], and breast cancer [37]. The top five highly expressed miRNAs in YST were miR‐122‐5p, miR‐200c‐3p, miR‐302a‐5p, hsa‐miR‐323a‐3p, and miR‐141‐3p. None of these miRNAs have been previously described in GCTs, but they play important roles in nephroblastoma [38], melanoma [39], lung tumors [40], and colorectal cancer [41]. In dysgerminomas, the top five overexpressed miRNAs were miR‐142‐3p, miR‐146a‐5p, miR‐21‐5p, miR‐182‐5p, and miR‐223‐3p. The upregulation of miR‐142‐3p [42], miR‐182‐5p [43], and miR‐223‐3p [44] has been previously shown in TGCTs. In addition, the increased expression of miR‐146a‐5p and miR‐21‐5p has been correlated with other cancer types, including breast [45], penile [46], and prostate [47], but not with GCTs.

We examined whether alterations in the miRNA signature were associated with the primary site of the tumor, we identified 20 miRNAs specific to testicular tumor and 18 miRNAs specific to ovarian tumors, and 31 differentially expressed miRNAs were in common between the ovary and testis. Among the 31 miRNAs, it included the six miRNAs upregulated in YST, EC, and dysgerminomas (miR‐302a‐3p, miR‐302b‐3p, miR‐367‐3p, miR‐371a‐5p, miR‐372‐3p, and miR‐373‐3p), suggesting that GCTs have similar histological pattern independent of their primary site [48].

The lack of longitudinal follow‐up in our study limits the analysis of miRNA levels in recurrent cases. In addition, we focused on gonadal tumors (ovarian and testicle) since it is more frequent and on five histological types (YST, dysgerminoma, EC, and mature and immature teratoma). We did not have samples to represent choriocarcinoma and seminoma histologies. We excluded mixed GCT samples because it is difficult to make a comprehensive evaluation of this tumor as a whole, and microdissection is more recommended. Therefore, further studies involving other primary tumor locations and histological types, including testicular seminoma, choriocarcinoma, and mixed GCTs, are needed.

The findings of our study are consistent with previous evidence pointing to miRNAs as potential biomarkers in pediatric GCTs. Our results indicate that the signature of miRNAs for each histological type may be useful as a standard clinical assessment to help with the diagnosis, prognosis, and management of GCT patients, but further studies are necessary to validate our findings.

## 5 Conclusions

We report that miRNA expression profiles are associated with histological types of pediatric gonadal GCTs, regardless of the primary site. Yolk sac tumor, dysgerminoma, embryonal carcinoma, and immature teratoma showed an overlap of these six miRNAs miR‐302b‐3p, miR‐302a‐3p, miR‐372‐3p, miR‐373‐3p, miR‐367‐3p, and miR‐371a‐5p, suggesting their clinical value for the management of pediatric patients with malignant GCTs. Our results highlight the differentially expressed miRNAs for each histological type, demonstrating the potential for microRNA expression to assist the diagnosis, prognostic, and management of patients with malignant GCTs.

## Conflict of interest

The authors declare no conflict of interest.

## Author contributions

AGSV: Conceptualization, Data curation, Formal analysis, Investigation, Methodology, Resources, Software, Visualization, Writing—original draft. LSS: Methodology, Software, Validation, Writing—review & editing. ECAS: Data curation, Methodology, Investigation, Writing—review & editing. ACL: Investigation, Methodology, Writing—review & editing. TMVF: Data curation, Writing—review & editing. AHL: Investigation, Methodology, Writing—review & editing. GEM: Conceptualization, Data curation, Writing—review & editing. MAO: Formal analysis, Methodology, Validation, Writing—review & editing. RMR: Funding acquisition, Investigation, Methodology, Resources, Validation, Writing—review & editing. LFL: Conceptualization, Investigation, Methodology, Project administration, Resources, Supervision, Validation, Writing—review & editing. MTP: Conceptualization, Formal analysis, Investigation, Methodology, Project administration, Resources, Supervision, Validation, Writing—original draft, Writing—review & editing.

### Peer review

The peer review history for this article is available at https://www.webofscience.com/api/gateway/wos/peer‐review/10.1002/1878‐0261.13617.

## Supporting information


**Fig. S1.** Heatmap and dendrogram of differentially expressed miRNAs in pediatric malignant germ cell tumors.
**Fig. S2.** MicroRNA expression profile of each germ cell tumors histology when compared with control samples.

**Table S1.** List of 34 differentially expressed miRNAs in dysgerminomas compared with healthy control samples.

**Table S2.** List of 13 differentially expressed miRNAs in embryonal carcinoma compared with healthy control samples.

**Table S3.** List of 25 differentially expressed miRNAs in yolk sac tumors compared to healthy control samples.

**Table S4.** Overlap of 31 differentially expressed miRNAs in testicular and ovarian tumors.

## Data Availability

The datasets analyzed in the current study are available from the corresponding author upon reasonable request.
