# Supplementary material for: Comprehensive microRNA expression analysis of pediatric gonadal germ cell tumors: unveiling novel biomarkers and signatures
Source: Mol Oncol. 2024 May 9;18(6):1593–607. doi: 10.1002/1878-0261.13617 (PMC11161733; doi:10.1002/1878-0261.13617)
Supplement: Supplementary file 1 — Fig. S1. Heatmap and dendrogram of differentially expressed miRNAs in pediatric malignant germ cell tumors. Fig. S2. MicroRNA expression profile of each germ cell tumors histology when compared with control samples. [file MOL2-18-1593-s002.zip › Figure Legends.docx]

**Supporting information**

**Supplementary Table 1.** List of 34 differentially expressed miRNAs in dysgerminomas compared with healthy control samples.

**Supplementary Table 2.** List of 13 differentially expressed miRNAs in embryonal carcinoma compared with healthy control samples.

**Supplementary Table 3.** List of 25 differentially expressed miRNAs in yolk sac tumors compared to healthy control samples.

**Supplementary Table 4.** Overlap of 31 differentially expressed miRNAs in testicular and ovarian tumors.

**Supplementary Figure 1. Heatmap and dendogram of differentially expressed miRNAs in pediatric malignant germ cell tumors.** Expression levels were used to group miRNA profiles according to their similarities between histological types, including embryonal carcinoma, dysgerminoma, yolk sac tumor, and mature and immature teratoma. The heatmap shows a distinct miRNA expression profile in the groups studied. Rows indicate the relative expression levels for a single miRNA, and columns indicate the expression level for a single sample. Blue and red colors indicate the miRNAs with lower and higher expression levels, respectively.

**Supplementary Figure 2. MicroRNA expression profile of each germ cell tumors histology when compared with control samples.** The heatmap shows a distinct miRNA expression profile in the dysgerminoma (A), yolk sac tumor (B), embryonal carcinoma (C), immature teratoma (D), and mature teratoma (E). Rows indicate the relative expression levels for a single miRNA, and columns indicate the expression level for a single sample. Blue and red colors indicate the miRNAs with lower and higher expression levels, respectively.
