# Supplementary figures and images for: Comprehensive microRNA expression analysis of pediatric gonadal germ cell tumors: unveiling novel biomarkers and signatures
Source: Mol Oncol. 2024 May 9;18(6):1593–607. doi: 10.1002/1878-0261.13617 (PMC11161733; doi:10.1002/1878-0261.13617)

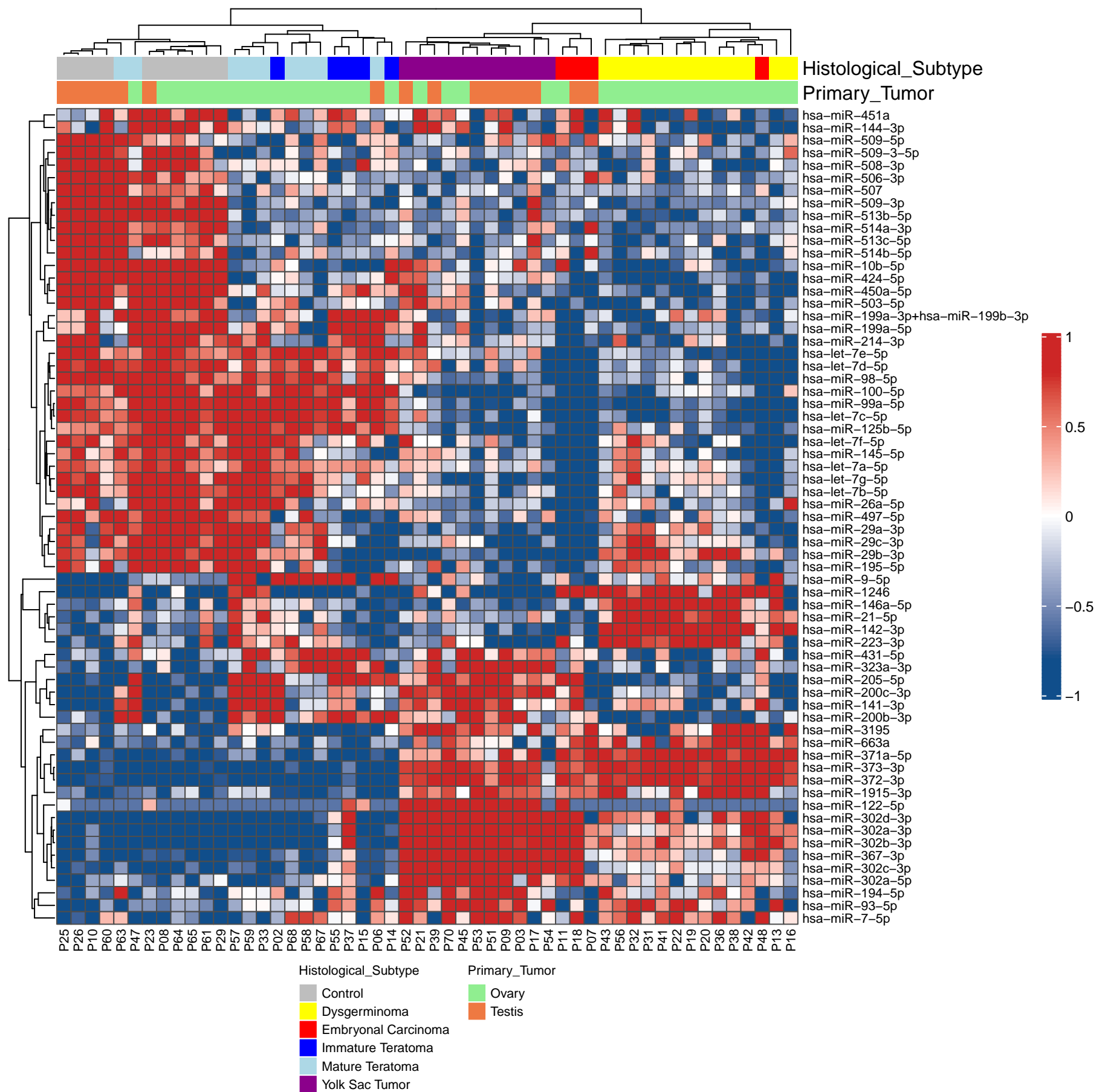

Supplement: Supplementary file 1 — Fig. S1. Heatmap and dendrogram of differentially expressed miRNAs in pediatric malignant germ cell tumors. Fig. S2. MicroRNA expression profile of each germ cell tumors histology when compared with control samples. [file MOL2-18-1593-s002.zip › mol213617-sup-0001-FigureS1.pdf]

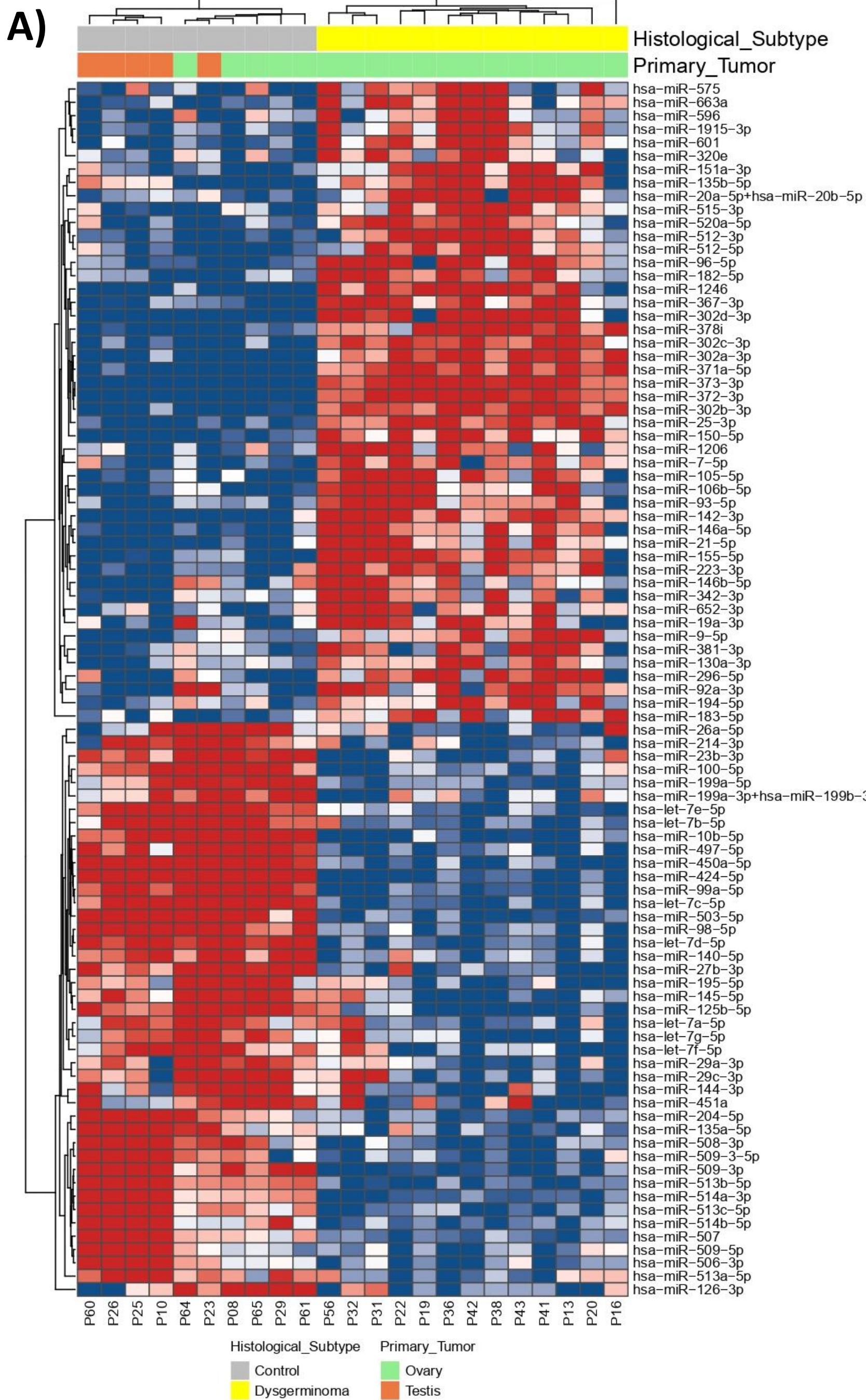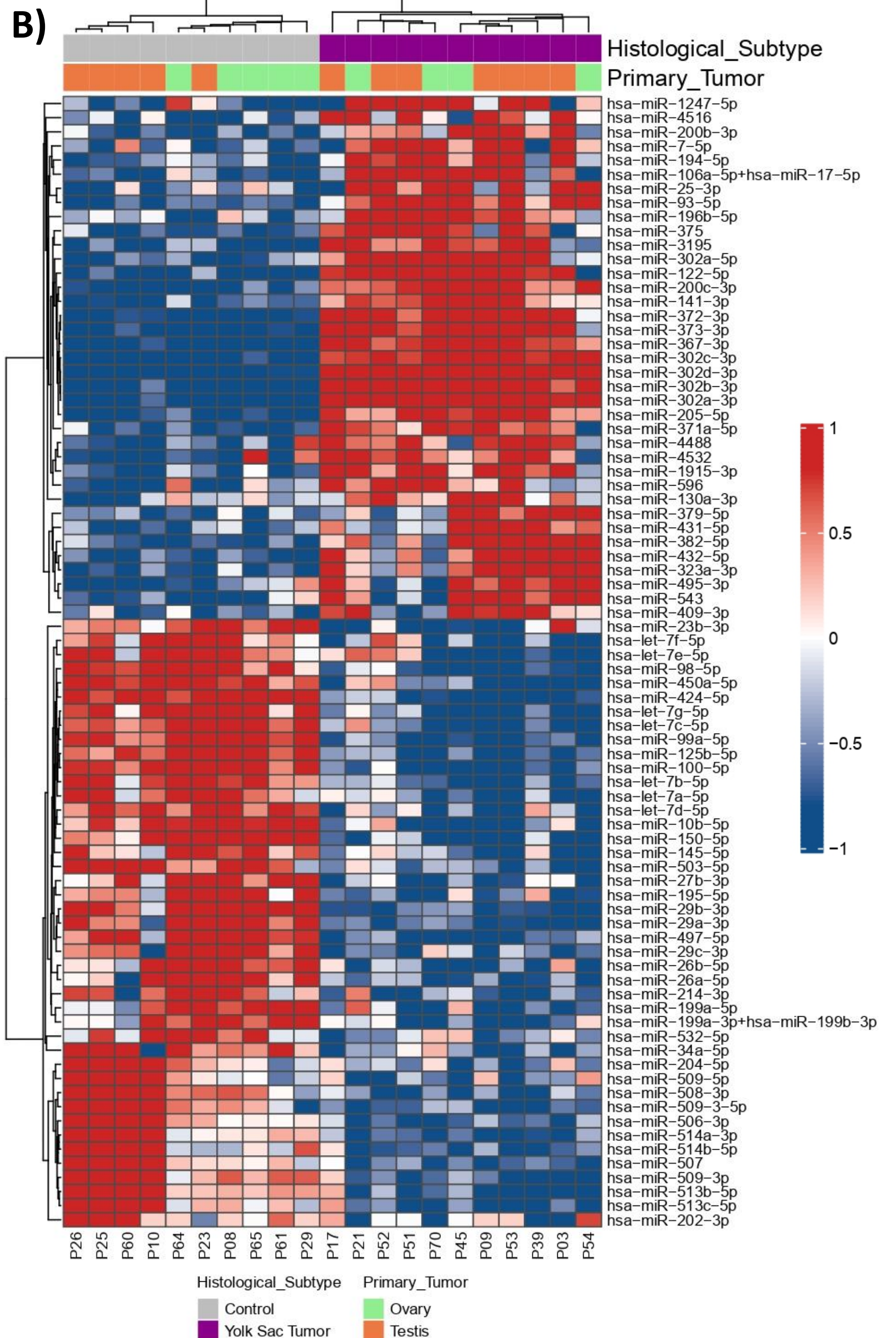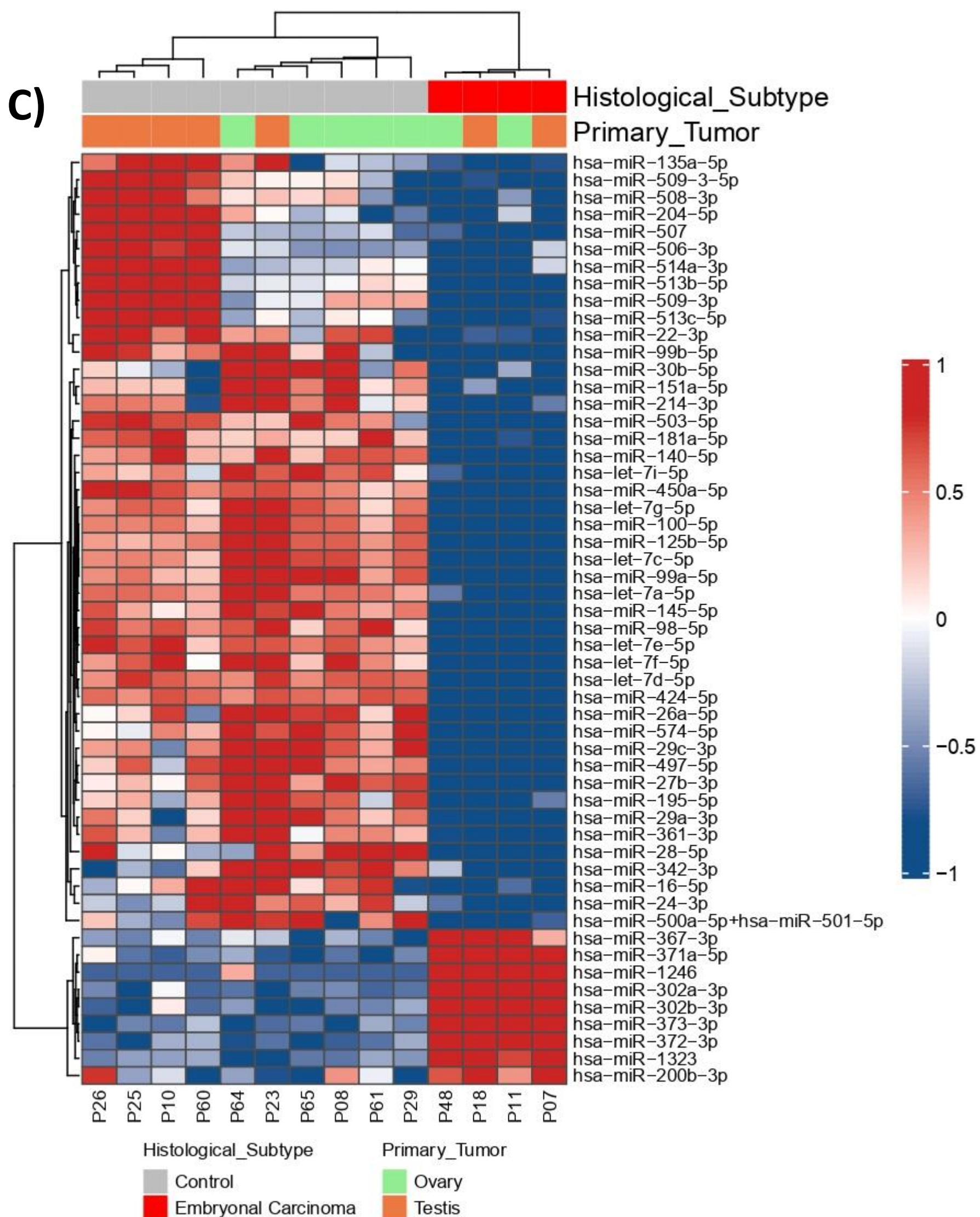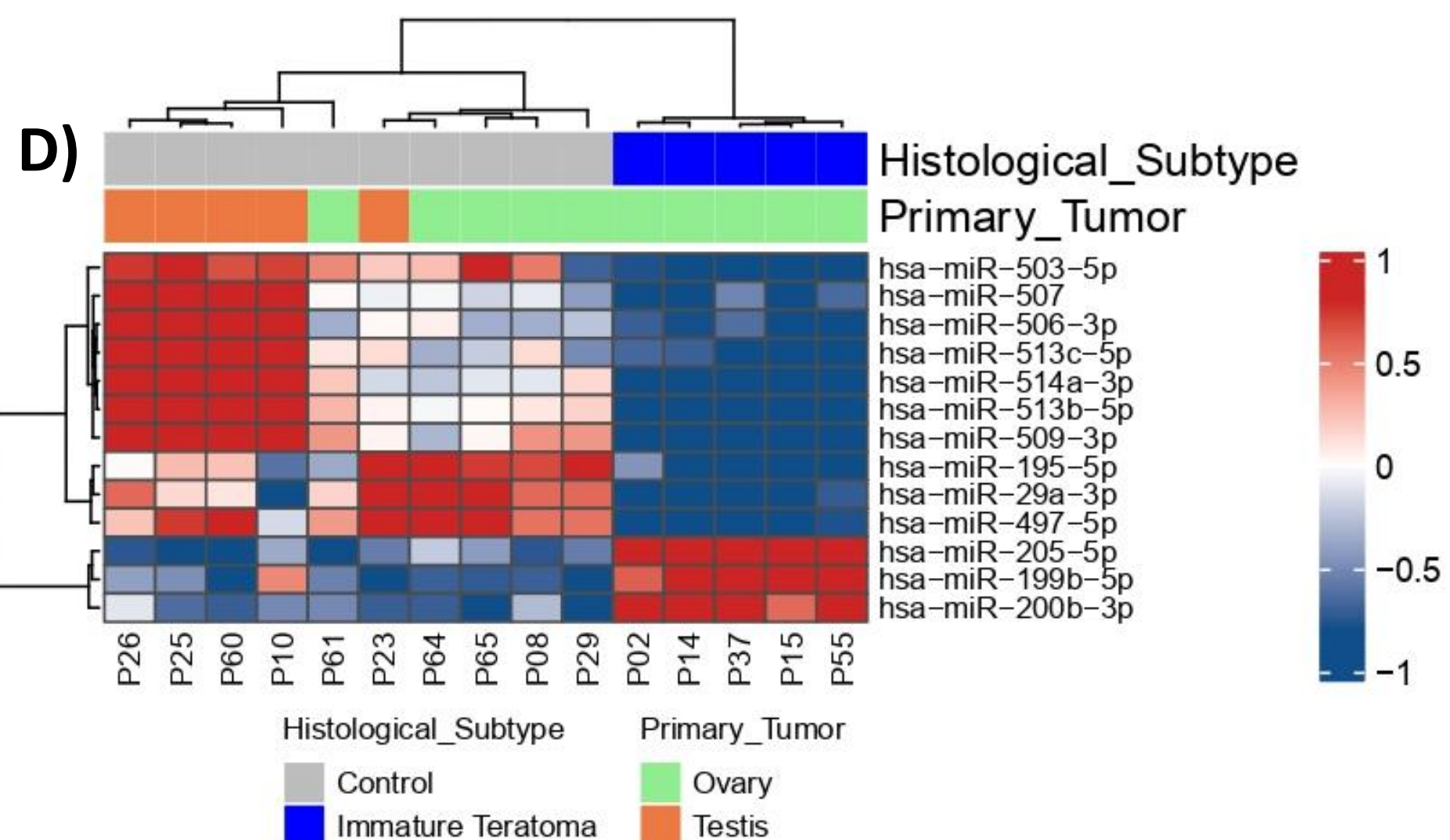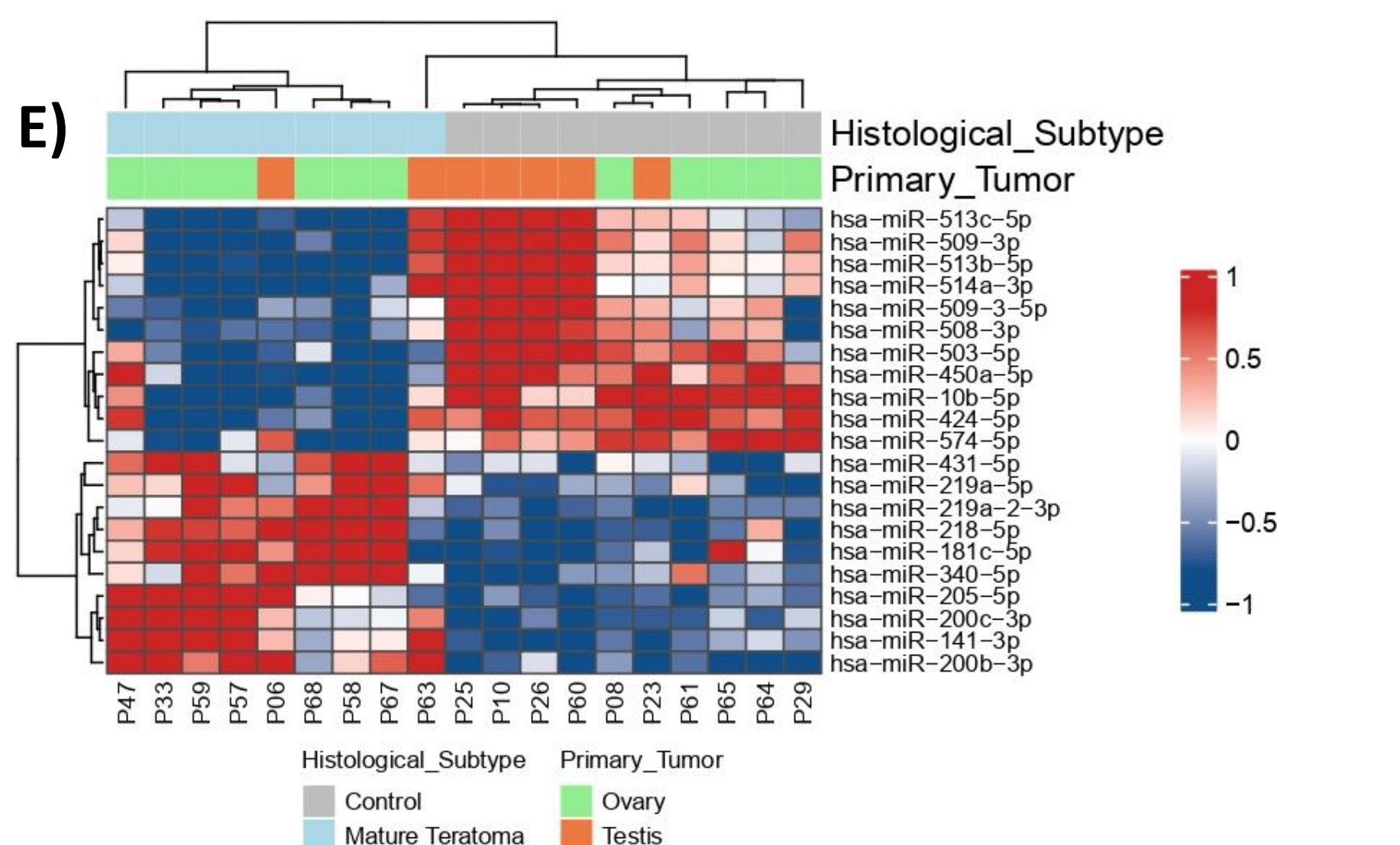

Supplement: Supplementary file 1 — Fig. S1. Heatmap and dendrogram of differentially expressed miRNAs in pediatric malignant germ cell tumors. Fig. S2. MicroRNA expression profile of each germ cell tumors histology when compared with control samples. [file MOL2-18-1593-s002.zip › mol213617-sup-0002-FigureS2.pdf]
