# Supplementary material for: Comprehensive microRNA expression analysis of pediatric gonadal germ cell tumors: unveiling novel biomarkers and signatures
Source: Mol Oncol. 2024 May 9;18(6):1593–607. doi: 10.1002/1878-0261.13617 (PMC11161733; doi:10.1002/1878-0261.13617)
Supplement: Supplementary file 2 — Table S1. List of 34 differentially expressed miRNAs in dysgerminomas compared with healthy control samples. [file MOL2-18-1593-s001.docx]

**Supplementary Table 1.** List of 34 differentially expressed miRNAs in dysgerminomas compared with healthy control samples.

| **miRNAs** | **p_adj** | **Log2 Fold Change** |
| --- | --- | --- |
| hsa-miR-142-3p | 0,00000013 | 3,8 |
| hsa-miR-146a-5p | 0,00026 | 2,8 |
| hsa-miR-21-5p | 0,0001 | 2,7 |
| hsa-miR-182-5p | 0,00068 | 2,4 |
| hsa-miR-223-3p | 0,00067 | 2,1 |
| hsa-miR-601 | 0,00018 | 2,1 |
| hsa-miR-135b-5p | 0,0045 | 2,1 |
| hsa-miR-9-5p | 0,0013 | 2,0 |
| hsa-miR-512-3p | 0,00048 | 1,9 |
| hsa-miR-663a | 0,00037 | 1,9 |
| hsa-miR-105-5p | 0,00037 | 1,8 |
| hsa-miR-155-5p | 0,000022 | 1,7 |
| hsa-miR-296-5p | 0,0052 | 1,6 |
| hsa-miR-515-3p | 0,00041 | 1,5 |
| hsa-miR-512-5p | 0,00036 | 1,4 |
| hsa-miR-96-5p | 0,000095 | 1,4 |
| hsa-miR-378i | 0,0000012 | 1,3 |
| hsa-miR-520a-5p | 0,000000045 | 1,3 |
| hsa-miR-106b-5p | 0,00026 | 1,2 |
| hsa-miR-151a-3p | 0,0013 | 1,2 |
| hsa-miR-92a-3p | 0,022 | 1,1 |
| hsa-miR-1206 | 0,013 | 1,0 |
| hsa-miR-146b-5p | 0,047 | 1,0 |
| hsa-miR-183-5p | 0,0042 | 1,0 |
| hsa-miR-19a-3p | 0,032 | 1,0 |
| hsa-miR-20a-5p+hsa-miR-20b-5p | 0,0045 | 1,0 |
| hsa-miR-320e | 0,047 | 1,0 |
| hsa-miR-381-3p | 0,026 | 1,0 |
| hsa-miR-575 | 0,012 | 1,0 |
| hsa-miR-652-3p | 0,0079 | 1,0 |
| hsa-miR-126-3p | 0,047 | -1,0 |
| hsa-miR-513a-5p | 0,0045 | -1,4 |
| hsa-miR-144-3p | 0,0046 | -1,5 |
| hsa-miR-451a | 0,033 | -1,8 |
