# Supplementary material for: Comprehensive microRNA expression analysis of pediatric gonadal germ cell tumors: unveiling novel biomarkers and signatures
Source: Mol Oncol. 2024 May 9;18(6):1593–607. doi: 10.1002/1878-0261.13617 (PMC11161733; doi:10.1002/1878-0261.13617)
Supplement: Supplementary file 3 — Table S2. List of 13 differentially expressed miRNAs in embryonal carcinoma compared with healthy control samples. [file MOL2-18-1593-s004.docx]

**Supplementary Table 2.** List of 13 differentially expressed miRNAs in embryonal carcinoma compared with healthy control samples.

| **miRNAs** | **p_adj** | **Log2 Fold Change** |
| --- | --- | --- |
| hsa-miR-1323 | 0,025 | 3,8 |
| hsa-miR-28-5p | 0,00027 | -1,1 |
| hsa-miR-24-3p | 0,011 | -1,2 |
| hsa-miR-361-3p | 0,0035 | -1,2 |
| hsa-miR-500a-5p+hsa-miR-501-5p | 0,006 | -1,2 |
| hsa-miR-16-5p | 0,006 | -1,3 |
| hsa-miR-574-5p | 0,0042 | -1,3 |
| hsa-miR-30b-5p | 0,021 | -1,5 |
| hsa-miR-151a-5p | 0,022 | -1,6 |
| hsa-miR-99b-5p | 0,0033 | -1,6 |
| hsa-miR-22-3p | 0,018 | -2,0 |
| hsa-miR-181a-5p | 0,04 | -2,4 |
| hsa-let-7i-5p | 0,029 | -3,0 |
