# Supplementary material for: Comprehensive microRNA expression analysis of pediatric gonadal germ cell tumors: unveiling novel biomarkers and signatures
Source: Mol Oncol. 2024 May 9;18(6):1593–607. doi: 10.1002/1878-0261.13617 (PMC11161733; doi:10.1002/1878-0261.13617)
Supplement: Supplementary file 4 — Table S3. List of 25 differentially expressed miRNAs in yolk sac tumors compared to healthy control samples. [file MOL2-18-1593-s003.docx]

**Supplementary Table 3.** List of 25 differentially expressed miRNAs in yolk sac tumors compared with healthy control samples.

| **miRNAs** | **p_adj** | **Log2 Fold Change** |
| --- | --- | --- |
| hsa-miR-122-5p | 0,000019 | 9,6 |
| hsa-miR-200c-3p | 0,000000084 | 3,6 |
| hsa-miR-302a-5p | 0,00025 | 2,4 |
| hsa-miR-323a-3p | 0,00025 | 2,3 |
| hsa-miR-141-3p | 0,000014 | 2,2 |
| hsa-miR-196b-5p | 0,0049 | 1,9 |
| hsa-miR-375 | 0,0017 | 1,8 |
| hsa-miR-432-5p | 0,00053 | 1,8 |
| hsa-miR-3195 | 0,00039 | 1,6 |
| hsa-miR-4488 | 0,0027 | 1,5 |
| hsa-miR-1247-5p | 0,031 | 1,4 |
| hsa-miR-431-5p | 0,0041 | 1,4 |
| hsa-miR-4516 | 0,0041 | 1,4 |
| hsa-miR-382-5p | 0,00092 | 1,3 |
| hsa-miR-4532 | 0,0035 | 1,3 |
| hsa-miR-543 | 0,0081 | 1,3 |
| hsa-miR-106a-5p+hsa-miR-17-5p | 0,002 | 1,1 |
| hsa-miR-409-3p | 0,02 | 1,1 |
| hsa-miR-495-3p | 0,008 | 1,1 |
| hsa-miR-379-5p | 0,0074 | 1,0 |
| hsa-miR-26b-5p | 0,00046 | -1,0 |
| hsa-miR-34a-5p | 0,0032 | -1,0 |
| hsa-miR-532-5p | 0,02 | -1,1 |
| hsa-miR-202-3p | 0,049 | -1,4 |
| hsa-miR-29b-3p | 0,0000012 | -2,9 |
