# Supplementary material for: Comprehensive microRNA expression analysis of pediatric gonadal germ cell tumors: unveiling novel biomarkers and signatures
Source: Mol Oncol. 2024 May 9;18(6):1593–607. doi: 10.1002/1878-0261.13617 (PMC11161733; doi:10.1002/1878-0261.13617)
Supplement: Supplementary file 5 — Table S4. Overlap of 31 differentially expressed miRNAs in testicular and ovarian tumors. [file MOL2-18-1593-s005.docx]

**Supplementary Table 4.** Overlap of 31 differentially expressed miRNAs in testicular and ovarian tumors.

|  | **Testis** | **Ovary** |
| --- | --- | --- |
| **miRNAs** | **Log2 Fold Change** | **Log2 Fold Change** |
| hsa-miR-302d-3p | 7,9 | 5,3 |
| hsa-miR-302b-3p | 7,4 | 4,8 |
| hsa-miR-302a-3p | 5,5 | 3,4 |
| hsa-miR-302c-3p | 5,1 | 2,7 |
| hsa-miR-372-3p | 4,9 | 4,5 |
| hsa-miR-373-3p | 4,9 | 4,3 |
| hsa-miR-367-3p | 3,9 | 3,4 |
| hsa-miR-205-5p | 3,5 | 2,9 |
| hsa-miR-200c-3p | 3,2 | 1,8 |
| hsa-miR-141-3p | 2,5 | 1,2 |
| hsa-miR-200b-3p | 1,6 | 1,1 |
| hsa-miR-371a-5p | 1,6 | 2,0 |
| hsa-let-7f-5p | -1,3 | -1,2 |
| hsa-miR-10b-5p | -1,3 | -1,9 |
| hsa-miR-98-5p | -1,4 | -1,1 |
| hsa-miR-497-5p | -1,5 | -1,8 |
| hsa-let-7d-5p | -1,7 | -1,3 |
| hsa-miR-503-5p | -1,7 | -1,8 |
| hsa-miR-29c-3p | -1,9 | -1,6 |
| hsa-miR-100-5p | -2,3 | -1,3 |
| hsa-miR-450a-5p | -2,4 | -2,2 |
| hsa-let-7b-5p | -2,6 | -1,3 |
| hsa-miR-29b-3p | -2,8 | -1,3 |
| hsa-let-7c-5p | -2,9 | -2,6 |
| hsa-miR-99a-5p | -3,1 | -2,9 |
| hsa-miR-125b-5p | -3,2 | -1,8 |
| hsa-miR-424-5p | -3,3 | -3,5 |
| hsa-miR-514a-3p | -4,2 | -2,4 |
| hsa-miR-513c-5p | -4,9 | -2,4 |
| hsa-miR-513b-5p | -5,3 | -3,5 |
| hsa-miR-509-3p | -5,7 | -4,0 |
